# Vascularized organ bioprinting: From strategy to paradigm

**DOI:** 10.1111/cpr.13453

**Published:** 2023-03-16

**Authors:** Heran Wang, Xin Liu, Qi Gu, Xiongfei Zheng

**Affiliations:** ^1^ State Key Laboratory of Robotics, Shenyang Institute of Automation Chinese Academy of Sciences Shenyang China; ^2^ Institutes for Robotics and Intelligent Manufacturing Chinese Academy of Sciences Shenyang China; ^3^ University of Chinese Academy of Sciences Beijing China; ^4^ State Key Laboratory of Membrane Biology, Institute of Zoology Chinese Academy of Sciences Beijing China

## Abstract

Over the past two decades, 3D bioprinting has become a popular research topic worldwide, as it is the most promising approach for manufacturing vascularized organs in vitro. However, transitioning from bioprinting of simple tissue models to real biomedical applications is still a challenge due to incomplete interdisciplinary theoretical knowledge and imperfect multi‐technology integration. This review examines the goals of vasculature manufacturing and proposes new strategic objectives in three stages. We then outline a bidirectional manufacturing strategy consisting of top‐down reconstruction (bioprinting) and bottom‐up regeneration (cellular behaviour). We also provide an in‐depth analysis of the four aspects of design, ink, printing and culture. Furthermore, we present the ‘construction‐comprehension cycle’ research paradigm and the ‘math‐model‐based batch insights generator’ research paradigm for the future, which may have the potential to revolutionize the biomedical field.

## Graphical abstract

This review examines the goals of vasculature manufacturing and suggests the objectives in three stages. Then, we outline a bidirectional manufacturing strategy through top‐down reproduction (bioprinting) and bottom‐up regeneration (cellular behaviour). Next, we introduce the views in detail from four links: design, ink, printing and culture. Furthermore, we present the ‘constructing‐comprehension cycle’ research paradigm in Strategic Priority Research Program (SPRP) and the ‘math‐model‐based batch insights generator’ research paradigm for the future with the potential to revolutionize the biomedical field. This review summarizes and distils the research results and ideas of the SPRP in the field of in vitro organ reconstruction and manufacturing. Unlike the common reviews in this field, this paper presents fresh ideas, highlights overlooked issues, corrects some misconceptions and points out future research directions.

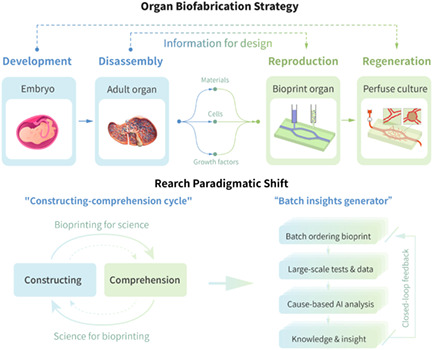

## 1 EXCITING GOAL: MANUFACTURING ORGANS IN VITRO

The in vitro manufacturing of human organs is anticipated to bring about a revolution in biomedical fields such as organ transplantation, drug development and pathophysiology emulation. Bioprinting has emerged as one of the most promising biofabrication^1^ strategies for tissue engineering and regenerative medicine,^2^, ^3^ owing to its capacity to precisely arrange cells and biomaterials in three‐dimensional space. Despite the remarkable advances in science and technology, the intricate nature of human organs still constitutes a major impediment to in vitro organ manufacturing.

### 1.1 Three‐step objectives

We propose three objectives for constructing human organs in vitro: (1) Short‐term goal (in ~5 years): creating vascularized, implantable, volumetric organs; (2) Long‐term goal (in ~15 years): producing functional, transplantable, full‐size organs; (3) Ultimate goal (in ~30 years): achieving clinical, patient‐matched, autogenous organs. Early bioprinting efforts with regards to microvasculature have focused on constructing a hollow lumen and forming an endothelial monolayer.^4^ Currently, scientists are further investigating the creation of vascular networks, that can supply nutrients and oxygen to volumetric tissues and anastomose with animal host vasculatures, while precisely distributing parenchymal tissue cells during bioprinting. With an increased understanding of organ regeneration and development, as well as the industrialization of bioprinting technology, it is possible to design multi‐scale structures based on individual patient requirements and manufacture transplantable organs on‐site in hospitals by utilizing the patient's autologous stem cells.

### 1.2 Organ‐level vascularization: the ‘Mars mission’ of bioengineering

#### 1.2.1 Complexity of organs

A total of about 80 types of human organs can be classified into four general levels by macrostructural complexity: flat, tubular, hollow and solid,^5^ of which solid organs are the most complex and representative. The solid organs are volumetric and multi‐scale in structure with multi‐tissue composition, multi‐cellular interactions and multi‐level tubular networks. From a reverse engineering standpoint, the biological organism is the most difficult to replicate due to their spontaneous emergence as a complex systems at many levels, such as tissues, multi‐cellular structural units, cells, organelles and biomolecular structures.^10^

#### 1.2.2 Microcirculation: a central objective in bionics

The key to sustaining volumetric tissue activity is to reconstruct the microcirculatory system, which is a network of arterioles (<0.3 mm), capillaries and venules (<0.2 mm). Vasculature reconstruction can be decomposed into three stages: vasculogenesis, angiogenesis and vascular remodelling. To facilitate this multicellular self‐organization process, biophysical and biochemical parameters, such as extracellular matrix (ECM) viscoplasticity, vascular endothelial growth factor (VEGF) gradients,^6^ oxygen content distribution, vascular wall shear stress (WSS), etc., and the interactions between multiple cell types, such vascular smooth muscle cells and pericytes, need to be regulated. This process regulates vital physiological functions such as endothelial barrier function, endothelial cell (EC) expansion and trans‐EC transportation.

#### 1.2.3 Vasculature from functional perspectives

Vascularization is ultimately necessary for achieving the functions of tissue oxygenation, nutrient delivery and waste disposal.^7^ Natural selection has tended to maximize both metabolic capacity (by maximizing surface area for exchange) and mechanical efficiency (by minimizing transport distances and time).^8^ In this case, organisms have evolved fractal hierarchical branching vascular networks that terminate in capillaries, which must eventually be located within ~200 μm of their target cells, depending on the maximum distance of diffusion of critical substances in vivo.^8^ Mimicking vasculature based on its functional goals, rather than blindly copying its hierarchical structure, is essential for our success in reconstructing it.

#### 1.2.4 Artificial vascularization

The reconstruction of vascularized tissues in vitro should aim to replicate natural conditions to the greatest extent possible, such as nutrient supply kinetics, blood flow mechanics and developmental dynamics. Nevertheless, natural vasculatures develop in a stage‐wise manner during embryogenesis, whereas artificial vasculatures must be able to provide nutrient supply immediately upon bioprinting. This challenge is akin to a Mars mission for bioengineering,^9^ possibly even more complex due to its intricacy at the micro‐scale. We suggest a 4‐level capability for vasculature morphology fabrication with increasing precision: (1) coarse simple planar branches (~1 mm); (2) fine, complex three‐dimensional networks (~0.5 mm); (3) dense fine‐grained endothelial networks (~250 μm); (4) volumetric microvascular network anastomosis with capillaries (~50 μm).

### 1.3 Dual deficit in scientific knowledge and bionic technologies

Although molecular and cell biology have seen rapid advances in recent decades, the complexity of multi‐biomolecular and multi‐cellular interactions at the tissue and organ levels, as well as the complex time‐dependent dynamics of development, still leave us with an inadequate understanding of the tissues and organs to be mimicked in bionics.^10^ Moreover, simply mimicking mature organ morphology may not be sufficient, and researchers are beginning to recognize the potential need to target earlier stages of organ development.^11^ Therefore, questions on how to reconstruct multi‐scale microenvironments and macrostructures have been challenging to answer.^12^ Current bionic and bioprinting technologies still cannot meet the necessary requirements for precision, efficiency and cytocompatibility simultaneously. Additionally, compared to the advances in innovative bioinks, innovations in bioprinting engineering and bionic techniques have been more challenging, particularly with regards to new hardware equipment and design software.

## 2 BIOPRINTING STRATEGY: RECONSTRUCTION AND REGENERATION

**FIGURE 1:**
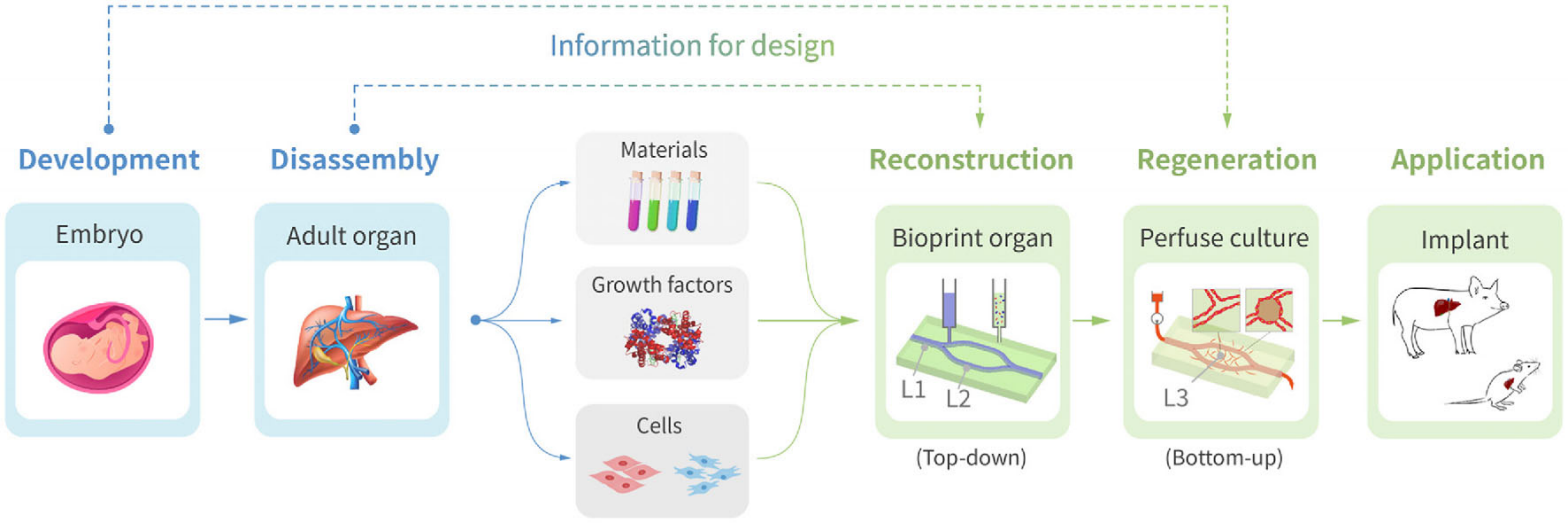
Schematic diagram of the RR framework for in vitro organ biofabrication, with a focus on information correspondence.

Our proposed bioprinting strategy for in vitro organ construction comprises two principal components (Figure 1): (1) Reconstruction (top‐down), which entails recreating the biophysical and biochemical forms of the cellular microenvironments and tissue macrostructures, through the assignment and assembly of bioinks in accordance with digital design; (2) Regeneration (bottom‐up), which focuses on nurturing the potential for self‐assembly during perfusion culture and digital monitoring, culminating in the self‐organization of tissue and organ morphology and function.

### 2.1 Bidirectional approach: top‐down and bottom‐up

Current reconstruction technologies (bioprinting or bioassembly) do not adequately replicate the complexity of organs (cross‐scale, hierarchical, geometric intricacy, microenvironment). To address this deficiency, our manufacturing strategy is to simulate the natural developmental intermediate state, and recreate the biophysical and biochemical conditions for cellular self‐organization. This empowers cells to autonomously complete the subsequent development. 2.5D structures in organs‐on‐chips, for example, the AngioChip^13^, ^14^ and other methods^15^ that follow this strategy are especially noteworthy.

The Reconstruction and Regeneration (RR) framework entails printing tissues with bioinks containing parenchymal cells, ECs, and other associated cells, printing arterioles and venules with sacrificial ink, perfusing with culture medium containing ECs to enable them to adhere to the vessel wall, stimulating EC tubularization, sprouting, and capillary anastomosis, and ultimately generating a functional, hierarchical microvasculature.^4^ Thus, we are putting forward a three‐level definition of engineered vessels for organ fabrication: L1 vessels are linked to culture tubes or animal hosts; L2 vessels constitute a multi‐branched network interconnecting inlet and outlet, supplying nutrients and oxygen to most cells; and L3 vessels constitute capillary networks brought about through EC tubulogenesis and sprouting angiogenesis.

#### 2.1.1 Top‐down reconstruction: 3D printing

**FIGURE 2:**
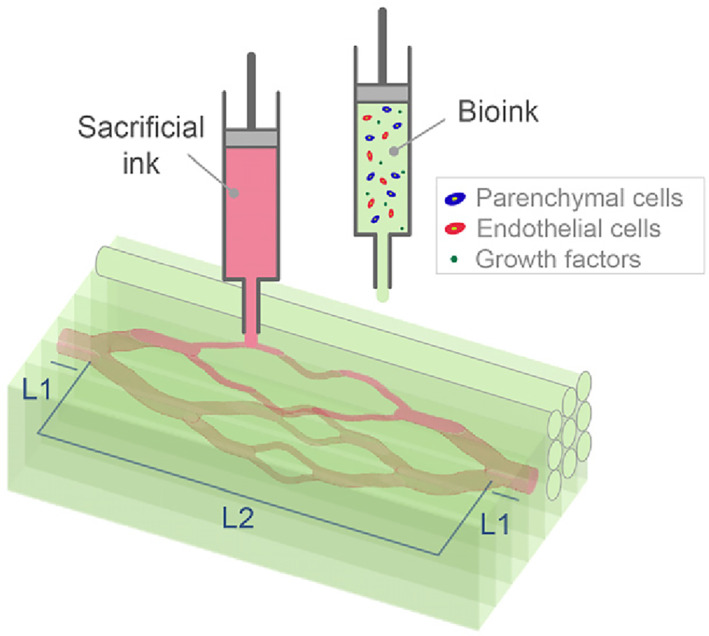
Schematic of a 3D bioprinting configuration.

Reconstruction (bioprinting, Figure 2) does not aim to completely mirror a mature organ, but instead to craft a regenerative environment with biophysical and biochemical cues to direct cell behaviour. Certain biological elements can be incorporated into the design of bioinks, such as cell sources, growth factors, cell adhesion ligands and mechanical properties. Geometric elements such as matrix fibre and signal distribution, and vascular topology, can also be printed by design. Interestingly, the lack of ability to print at capillary‐scale resolution is often viewed as a crucial hurdle. Nevertheless, capillaries cannot be constructed through direct printing since even if a 10‐micron tube could be printed, ECs with a similar diameter would be unable to perfuse it to effect vessel wall endothelialization. What then is the minimum bioprinting precision needed to reconstruct a well‐functioning, phenotypically accurate and reproducible organ system? We posit that the smallest printable duct should be at least several times the diameter of the ECs, thus the minimum ‘sufficient resolution’ for bioprinting is approximated to be 50 μm. Additionally, it is noteworthy that cells suspended in bioinks are nearly spherical in shape, while after bioprinting and growth, they gradually differentiate and flatten into the ‘ultimate state’.

#### 2.1.2 Bottom‐up regeneration: cellular self‐organization

**FIGURE 3:**
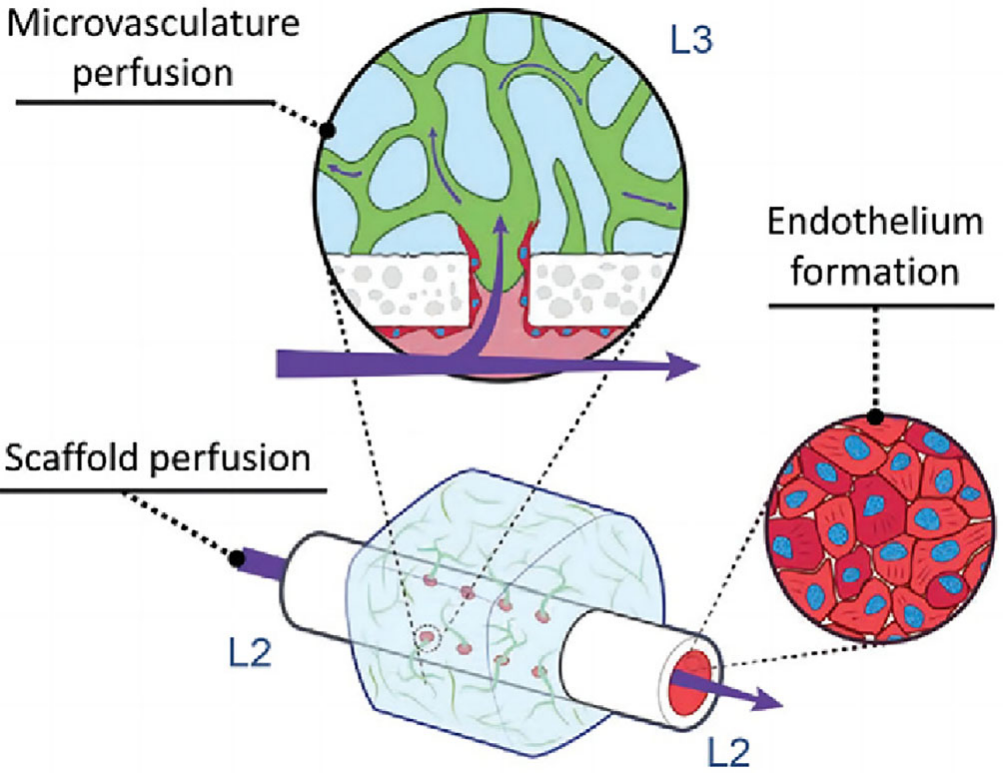
Schematic of bottom‐up cellular self‐organization. Reproduced with permission.^16^ Copyright 2021, WILEY.

From as early as the design stage, we should consider the dynamic conditions needed for regeneration, such as growth factor sustained‐release, oxygen gradients, morphogenesis, blood flow, etc. Nevertheless, many biological issues remain to be explored and understood; thus, the current rule‐of‐thumb is to remain as close as feasible to the in vivo environment. Throughout perfusion culture, cells autonomously respond to the surrounding mechanical and chemical environment to generate tissue‐level morphogenesis, such as vasculogenesis and angiogenesis (Figure 3). For complex biosystems, we must employ devices equipped with quantitative detection tools to monitor and control all relevant parameters. An analogy may be used to comprehend the dynamical control relationships between the in vitro culture device and the cultured tissue: the in vitro culture device is analogous to the pregnant mother, the cultured tissue is analogous to the foetus, and when the tissue is thoroughly developed and ready to be used in vivo, it is comparable to the birth of the foetus.

### 2.2 Design for vascularized tissues/organs

Design processes function as a compass for organ manufacturing, deciding the tissue's ultimate biological function. Nevertheless, current design research predominantly concentrates on simple geometric morphological sketching. We posit that bioprinting is entering a function‐oriented and model‐based designable phase, which could have a great impact on biofabrication.

#### 2.2.1 Biophysical models

**FIGURE 4:**
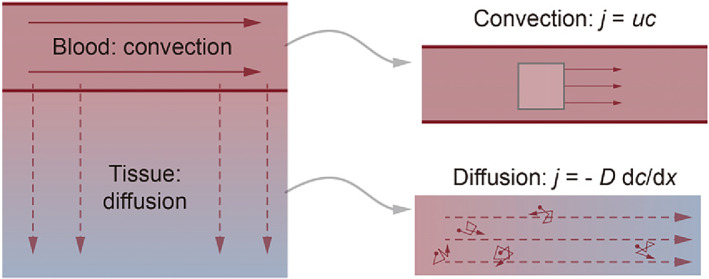
Basic principles of solute transport to tissue.

**FIGURE 5:**
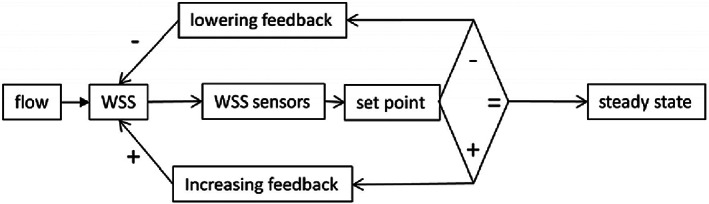
Classical WSS set point theory according to the concepts of control theory. Reproduced with permission.^22^ Copyright 2020, Frontiers.

**FIGURE 6:**
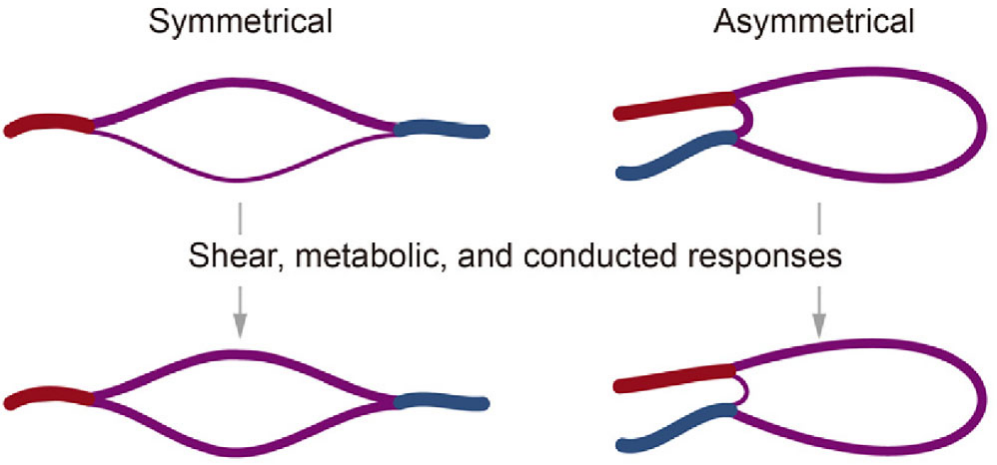
Responses to stimuli on microvascular diameters.

1. Substance diffusion model.^17^ The nutrient exchange functionality of vasculature relies on diffusive and convective solute transport (Figure 4). Recently, a parametric characterization based on the metabolically active (Krogh) radius has been unearthed,^18^ which is a comprehensive index combining the impacts of cellular matrix permeability, cell density and metabolic intensity. Literature frequently references capillaries with a maximum distance,^5^ which is practically twice the Krogh radius; however, in vitro organs possess superior matrix permeability, lower cell density and lower cellular metabolic intensity compared to in vivo, resulting in an enlarged Krogh radius, which theoretically denotes the design basis for the vascular network density of in vitro tissues. Given that nutrient exchange is situated at the vessel surface, we propose that the ‘vascular surface area’ coupled with the ‘vascular surface area per parenchymal tissue unit’ quantifies the functional‐oriented geometric traits of the vasculature. Moreover, the design of concentration gradient fields of biochemical molecules can be computed and simulated based on the reaction‐diffusion model, which can be referred to as Turing pattern related studies.^19^
2. Hemodynamic model.^20^ Vascular networks that are not hemodynamically compatible are susceptible to thrombotic issues since blood clotting is sensitive to the mechanical state of the vasculature.^21^ For example, rough vessel wall surfaces and non‐streamlined ducts can lead to turbulent flow, creating high local shear stresses and prompting a platelet clotting reaction. Murray's law, derived from the principle of minimum action in mechanics, is a beneficial guide for the structural design of branches, and has yielded the vessel wall shear stress (WSS) set point theory (SPT, Figure 5). The forces that blood flow exerts on the vasculature affect cellular behaviour, such as EC sensitivity to WSS, and SMC sensitivity to circumferential tensile stress, resulting in transformations in the vasculature's short‐ and long‐term morphology.^22^
3. Vascular development model. Microvascular remodelling adheres to the WSS SPT, and ECs typically behave as WSS sensors (sensor‐pathway model and tensegrity model),^22^, ^23^ which tend to adjust vessel diameter to maintain a stable level of pressure and WSS. Simultaneously, upstream and downstream responses must also be considered in order to finish a computable vascular development model (Figure 6).^20^ Naturally, the vascular development process can be computationally simulated through building mathematical models to comprehend these biological mechanisms and form an automated vasculature design algorithm.

#### 2.2.2 Design methodology

**FIGURE 7:**
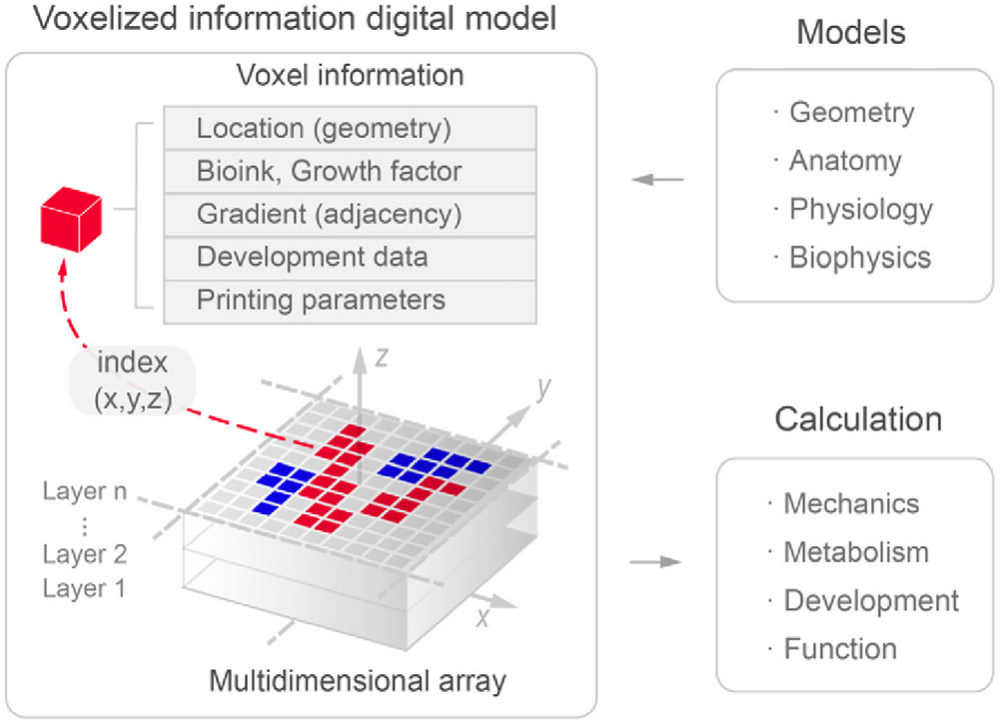
Voxelized multidimensional information digital model for organ (in vitro) design. Reproduced with permission.^24^ Copyright 2021, IOP.

Organ design is markedly different from traditional industrial design due to its information‐richness in three‐dimensional space. To accommodate the 3D printing process as well as the dynamic computable specifications, we propose that the model foundation for organ design should be a voxelized multidimensional information digital model (Figure 7).^24^ We predict that the philosophy of organ design will gradually transition from simple to complex systems.^28^ Thus we should formulate biophysical equations and cellular behaviour models based on biological principles, by using straightforward algorithmic rules to simulate and calculate tissue patterns.^25^ In this way, the design methodology will evolve from principle‐based to model‐based, from static analysis to dynamic simulation, and from ‘structure‐oriented’ to ‘structure‐function integration oriented’. It is foreseeable that model‐based computable digital designs will propel the field of in vitro organ manufacturing to become more scientific and inspiring.

### 2.3 Bioprinting inks for vascularized tissue

Bioprinting inks encompass bioinks and biomaterial inks (mainly assistive materials, including sacrificial inks and support baths^26^); the distinction lies in whether they contain cells.^27^ Much literature has been generated regarding the development of bioprinting inks; nevertheless, the material properties that are essential for fabricating cell‐material constructs that accurately imitate biological function, remain indeterminate.^28^ In addition, the R&D on biomaterials is largely search‐based rather than function‐oriented, drawing inspiration from ECM, food additives, cosmetics, or even industrial products to attain novel properties through blending and modifying.

#### 2.3.1 Bioink material

Bioink materials should not only possess fundamental properties such as printability, crosslinkability, structural stability, cytocompatibility and cell blendability, but also properties that stimulate cellular behavior.^5^ Cellular behaviours (e.g., migration) can be contingent on ECM viscoplasticity (i.e., viscosity,^29^ elasticity and plasticity), which is a near‐universal mechanical feature that requires an understanding of porosity, degradation,^30^ and dynamics, and which is indispensable for the replication of human tissue properties.^31^ Moreover, the local properties rather than the global properties of the material are pertinent to the cell‐material interaction behaviour, which necessitates increased focus on the sophisticated structure of natural tissue ECM.

**FIGURE 8:**
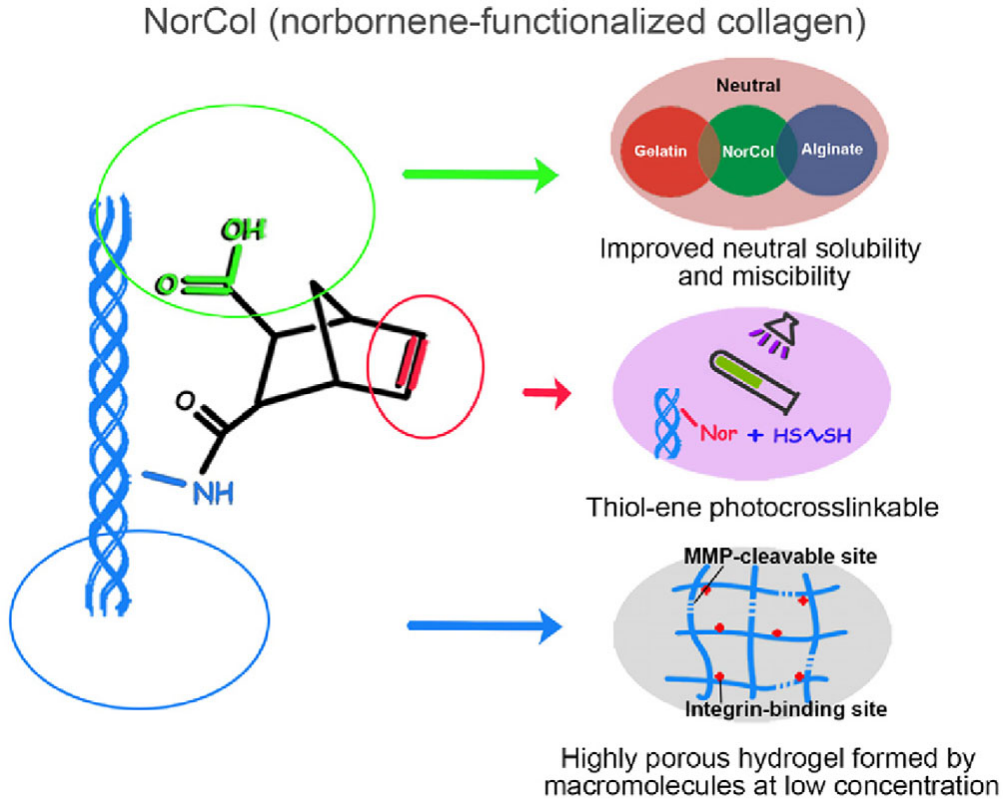
NorCol (norbornene‐functionalized collagen), a typical bioink. Reproduced with permission.^39^ Copyright 2021, ACS.

Bioinks are primarily natural materials (Table 1) or even decellularized extracellular matrix (dECM),^32^ complete with cell adhesion ligands,^33^ natural signalling capabilities and mechanical characteristics similar to those in vivo (Figure 8). Nevertheless, synthetic materials, such as PEG (polyethylene glycol), also have immense potential owing to their stability, programmability,^34^ and medical availability, especially if we can fully uncover the target properties of matrix materials through reductionism.^4^ Furthermore, after printing, the bioink can be cross‐linked physically or chemically,^35^ to obtain a microscopic network, which can significantly and programmably influence cellular behaviour.

**TABLE 1:** Bioink materials.

Base material	Polymer system	Crosslinking method	Ref.
Agarose	Carboxylated agarose	Temperature	36
Alginate	Alginate	Ionic crosslinking (Ca)	37
Collagen	Collagen	Glutaraldehyde	38
Collagen	NorCol	Thiol‐ene photoclick	39
Gelatin	GelMA	UV polymerization	40
Hyaluronic acid	HA‐methacrylate	UV polymerization	41
Fibrin	Fibrinogen	Temperature + ionic	42
Matrigel	Matrigel	Temperature	43
Silk	Silk/PEG	Temperature	44

#### 2.3.2 Cells in bioink

The majority of tissues comprise a variety of functional and supporting cells. In addition to the requisite functional cells, tissues also contain cell types that provide support, perform structural or barrier functions, form blood vessels, or support the maintenance or differentiation of stem cells.^45^ Presently, somatic cell printing is the most popular method utilizing bioinks, incorporating a variety of terminally differentiated somatic cells. (1) Adult cells of autologous or allogeneic origin extracted as primary cells from the tissue or organ of interest predominate. (2) Stem cells. Although most of the cells currently utilized in clinical trials are mesenchymal stem cells, utilizing pluripotent stem cells or tissue‐specific adult stem cells tailored to patient needs will be the trajectory of the future.^46^, ^47^

The cell types employed in bioinks must be able to replicate the target cell types various functions and be expanded in vitro to large‐scale organ printing quantities.^48^ For long‐term applications, the printed cells must adapt to all physiological conditions, such as shear stress, enzymes, etc., in culture or during use.^49^ They must also be resilient enough to endure the printing process or have sufficient proliferative capacity to preserve cell numbers through self‐renewal. It is essential to recognize that the incorporation of cells modifies the original ink's properties during printing, including modulus decreases, rheological characteristics changes, phase transition temperature changes, etc. Organoids or cell spheroids have also been utilized in bioinks in recent research, as the organoids are already functional building blocks.

In particular, induced pluripotent stem cells^50^ or clinical‐grade embryonic stem cells^51^ can serve as inks or sources of inks to solve the problem of clinical patient genetic matching. Stem cells, with their capacity for growth and potential for guided differentiation, compared to the limited capacity of mature somatic cells, are predicted to address the cell quantity issue at the root.^52^

#### 2.3.3 Sacrificial inks

Sacrificial or fugitive inks were introduced in the 2010s to sustain vessels throughout printing process and subsequently removed. The sacrifice mechanism typically includes aqueous dissolution, thermal gelation and melting and physical crosslinking disruption (Table 2). In bioprinting, the essential requirement for sacrificial inks is excellent printability and cell‐compatible removability. There is also another classification for the use of sacrificial inks: (1) support materials, that is, as external auxiliary supports for non‐regular structures with very low removal requirements or even manual peeling; (2) soluble core materials, which are sacrificial inks specifically designed to print microvascular networks thus require excellent removability, preferably in a phase change to liquid; (3) reinforcing materials, as components to temporarily improve the printability of bioinks; and (4) porosifier materials, which serve as phase separation components that produce cell‐friendly pores for bioinks after removal.^53^, ^54^, ^55^

**TABLE 2:** Sacrificial inks.

Removal mechanism	Material examples	Advantages	Disadvantages	Ref.
Aqueous dissolution	Carbohydrate‐glass	Fast dissolution; smooth surface	High‐temperature printing; need polymer coating	56
	Isomalt sugar power	Fast dissolution	Only available for selective laser‐sintering (SLS)	18
	Laponite	Excellent formability	Slow dissolution	‐
Thermal melting	Pluronic F‐127	Stable properties; room temperature	Poor adhesion with some inks	57
	Gelatin	Melt at 37°C; naturally cell‐adhesive	Poor stability (variation over time)	
Molecular disruption	Alginate (ionic crosslinking)	Crosslinks disrupted by calcium chelators	Slow de‐gelling speed	59

#### 2.3.4 Support baths

Support baths (i.e., suspension media) have become a research hotspot in bioprinting since around 2015.^63^ Support baths are commonly yield‐stress and self‐healing materials, including gel‐phase and microparticle, that providing physical confinement during printing to improve resolution and shape fidelity (Table 3). Self‐healing means recovering at an appropriate rate after deformation by stress.

**TABLE 3:** Support baths.

Bath form	Bath material	Matching ink	Removal method	Features	Ref.
Gel‐phase	Laponite	Alginate/gelatin (cell)	Washed with NaCl	Simple material preparation; slow removal	60
	Pluronic F‐127‐DA	Pluronic F‐127	Low temperature (4°C)	Structurally stable; cell‐free; fast removal	61
Microparticle	Alginate microgel	Cell‐only ink	Washed with water	Direct cellular printing; average precision	62
	Carbopol granules	Polydi‐methylsiloxane (PDMS)	Washed with water	high precision; able to print cells	63
	Cell spheroids	Gelatin	Not removed (main body)	High cell density; easy removal	58
	Gelatin microparticles	Alginate and collagen	High temperature (37°C)	Realize collagen printing; cell‐friendly removal	38

### 2.4 Printing: key to complex organ fabrication

Bioprinting techniques for volumetrically sophisticated and heterogeneous tissue structures must precisely and accurately regulate soft matter inks and guarantee cellular activity and functional capacity throughout the procedure. As a universal biomanufacturing technology, 3D printing largely focuses on the development of solid tissues, and is also compatible with other simpler forms of tissues. Notably, our expectations on bioprinting are polarized: on the one hand, the capabilities of bioprinting are grossly overstated and often regarded as a one‐size‐fits‐all manufacturing solution, while on the other hand, the potential of bioprinting is often regarded as far from being fully explored and some technical obstacles are currently considered to be insurmountable.

#### 2.4.1 Mechanical process of printing

Printing is the process of assembling ink in 3D space as designed. This involves two key mechanical processes: material ‘transport’ and ‘assembly’. ‘Transport’ is the regulated movement of materials under the influence of forces, while ‘assembly’ is the combining of discrete materials. The accuracy and precision of these processes decide how closely the print outcomes coincide with the design.

**FIGURE 9:**
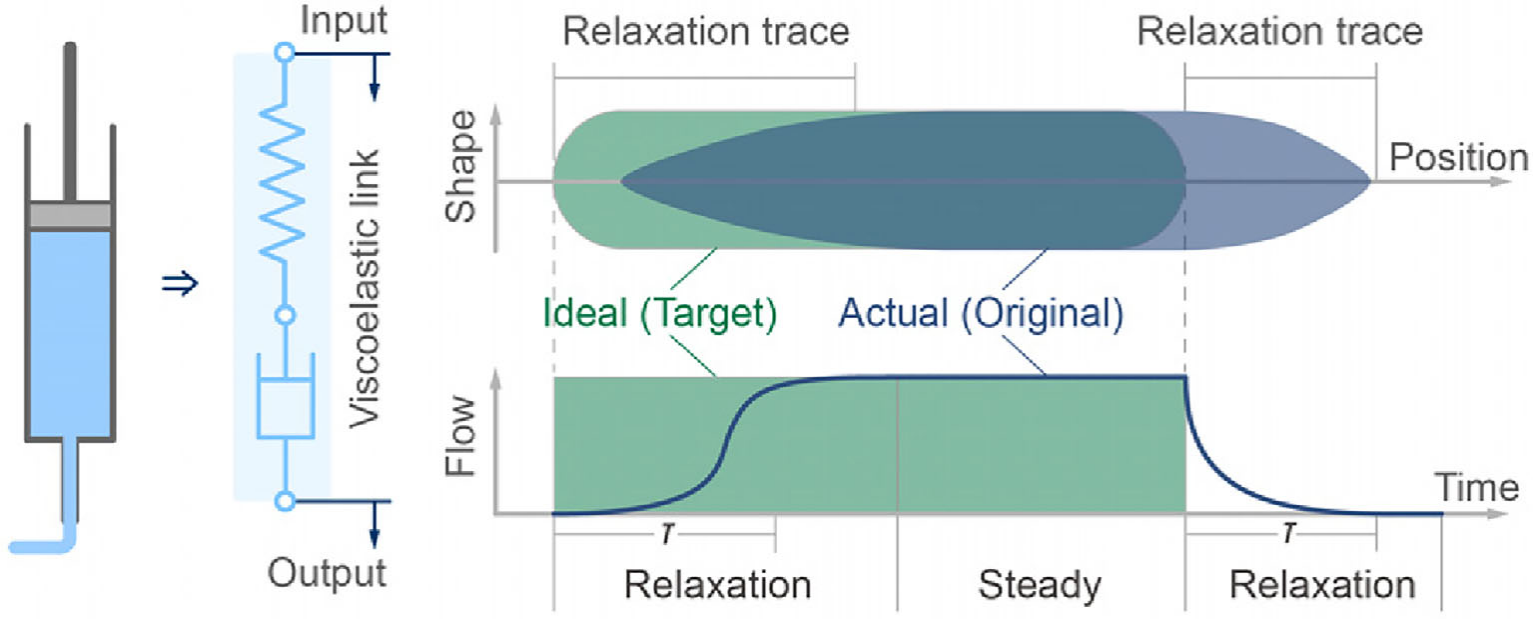
Schematic of the relaxation process in transport.

Mass transport is the consequence of a combination of factors associated with energy sources and flow channels. This mechanical perspective can enhance our comprehension of various printing approaches. For instance, the pneumatic printing type cannot be volumetrically dosed, and the nozzle tends to experience permanent blockage with poorly homogenized materials or agglomerated cells. Conversely, the electric piston type is volume‐controlled, and the pressure rises when obstruction occurs, thus automatically de‐clogging the nozzle. The term ‘transport precision’ alludes to the volume discrepancy between actual output and intended output, which is the primary concern in transport; especially when faced with the vast amount of starts and stops caused by the geometric complexity of a 3D hierarchical vascular network. We propose that this dynamic process should be viewed as a relaxation phenomenon (Figure 9), which can be quantitatively characterized by the ‘transport relaxation time *τ*’.

Assembly is the merging process of discrete materials, homogeneous or heterogeneous, when the old surface vanishes and a fresh surface appears. The ultimate morphology layered on the pre‐process structure depends on (1) the material self‐supportability, which is jointly determined by yield stress, viscosity and surface tension, and (2) the bonding and infiltration between materials and the pre‐process material properties, which is determined by interfacial tension, physical diffusion and chemical reaction; and the material relaxation time determines the dynamic processes. The assembly requirements of the materials vary depending on their purpose, for example, the vascular soluble core materials, should be sufficiently supported but not excessively infiltrated with native materials.

#### 2.4.2 Bioprinting approaches

Currently, bioprinting is classified into nozzle‐based and light‐based categories,^64^ where we can divide nozzle‐based into transport‐featured and assembly‐featured categories according to mechanical characteristics (Table 4). Generally speaking, the nozzle‐based method focuses on material dispersion yet needs formation precision, whereas the light‐based method brings good accuracy through a high‐resolution laser or digital micromirror device (DMD) but is challenging for multi‐material distribution.^65^

**TABLE 4:** Bioprinting approaches.

Categories	Approach names	Advantages	Disadvantages	Possible developments	Ref.
Nozzle‐based (transport‐featured)	Pneumatic extrusion	Simple equipment; disposable cartridge	Poor accuracy; prone to clogging	Arrayed efficient printing	66
	Electric piston extrusion	Volume control; good repeatability	Complex model; difficult to feed ink	Combine with microfluidic	67
	Electric screw extrusion	High viscosity; continuous feed	Rough control; hard to clean	Replaceable part	68
	Progressive cavity pump	Continuous feed; volumetric control	Complicated structure	Miniaturization; arraying	69
	Thermal inkjet	Fast printing speed; low equipment cost	Poor stability; clogging prone	Optimization of design	70
	Piezoelectric inkjet	Highly controllable; accurate positioning	Average cell friendliness	Simulation; force control	71
	Mirco‐valve inkjet	Simple equipment; wide viscosity range	Low resolution; High shear force	Smaller nozzle and size	72
	Acoustic inkjet	Very wide viscosity range; easy control	Difficult arraying; complex model	High‐density arrays	73
Nozzle‐based (assembly‐featured)	Embedded printing	High precision; flexible trajectory	Restricted volume; cumbersome	Structure form optimization	38
	Co‐axial printing	Suitable for tube; Rapid chemical reaction	Not for network; poor resolution	Coaxial flow focusing	74
	Microfluidic nozzle	Multi‐material switch; pre‐assembly	Cross‐contaminate; coarse nozzle	Gradient; high throughput	24
	Cell spheroid printing	Pre‐existing biological function	Restricted accuracy and precision	Ultra‐small cell spheres	75
Light‐based	Laser‐induced forward transfer	Highly accurate; medium‐speed method	Complex setup; Restricted height	Affordable; accessible	76
	Multi‐vat‐photopolymerization	Alter resin vat for multi‐material	Destructive cleaning; slow speed	Improve design concept	77
	Sequential injection (vat)	Rapid ink exchange; less consumption	Discarded hydrogel; limited area	Optimize cleaning method	78
	Sequential deposition (vat)	Bottom‐up DLP; faster; air‐jet cleaning	Contamination; deformation	Optimize cleaning and motion	79
	Volumetric/holographic printing	High speed; layerless; no harmful stress	Unable to achieve multi‐material	Multi‐material approach	80

Among all nozzle‐based techniques, extrusion^81^, ^82^ is the most widely used, cost‐effective, straightforward and convenient, with extensive applicability for inks with various viscosities, crosslinks and cell contents.^83^ Nevertheless, extrusion also confronts problems of low throughput, high shear stress and limited resolution. Drop‐on‐demand (DOD) inkjet^84^, ^85^, ^86^ has a high resolution, rapid speed, array integration and established commercial applications, but it is confined to lower viscosity inks and is only available for 2.5D structures. A handful of nozzle‐based methods (especially extrusion) have been tested to synergize with the cellular self‐assembly capacity for biological applications.^28^ (1) Pre‐assembly or pre‐setting^87^ of controlled material interfaces can be achieved by designing flow channels, such as a co‐axial nozzle and microfluidic channels. Coaxial printing has advantages in manufacturing single‐pipe structures and many applications, but it cannot handle the complex topology of multi‐branch vascular networks. (2) Embedded printing^38^ has received particular attention recently to obtain lower interfacial tension, cell‐friendly aqueous phase environment, and good morphology after diffusion in a self‐healing gel‐phase or microparticle support bath (with cell spheroids). (3) Cell spheroid printing combines microscopic cell self‐assembly with macroscopic distribution assembly using pre‐generated capillary networks of cell spheroids,^88^ organoids^89^, ^90^ and assembloids.^91^, ^92^

Light‐based printing achieves high‐precision moulding through an exemplary distribution of light/laser. Early stereolithography (SLA) methods utilized a micro spot to scan and cure quickly. When DMD emerged, ‘space for time’ was realized, thus significantly improving printing efficiency, and many innovative methods appeared employing digital light processing (DLP). The latest volumetric/holographic printing is undoubtedly high‐speed; yet, like DLP methods, the multi‐material distribution is still challenging. Some methods, such as multi‐vat‐photopolymerization, sequential injection, and sequential deposition, enable multi‐material printing to a certain extent. Nevertheless, frequent switching and cleaning limit efficiency and precision severely.

#### 2.4.3 Issues and developments of printing

Under the premise of multi‐material distribution,^93^ bioprinting engineering today confronts a triple paradox: precision, speed and cytocompatibility. Precision is the most concerning issue for users because actual tissue heterogeneity often takes place at a scale lower than printers can achieve. It should be noted that the actual precision is dissimilar from the machine's declared precision. Printing result fidelity and minimum feature size should be taken into account as co‐criteria. In addition, high precision often leads to slow speed, which poses a challenge for large‐volume printing and cell activity assurance. We use ‘ink volume flow rate’ to characterize the printing speed, but note that the auxiliary action time must be accounted for, as this is a long‐time session in some approaches. Finally, precise and fast methods generally result in poor cytocompatibility, often characterized by viability and functional protein secretion due to mechanical processes and ink properties.

3D bioprinting is widely expected to achieve accurate and rapid reproduction of designs, just as 2D commercial printers did, and there are three directions for future technological development. (1) New mechanisms: From the perspective of transport and assembly mechanics, novel printing concepts should be proposed to resolve the ‘precision‐speed‐cytocompatibility’ paradox. (2) Universality: Present approaches and materials are often restricted in terms of their scope of applications, not taking full advantage of their broad potential. (3) Miniaturization: In response to the growing cell density of bioinks and the preciousness of high‐tech materials, highly integrated micro‐pipetting systems should be developed to optimize ink consumption.

### 2.5 Culture: regeneration and application

**FIGURE 10:**
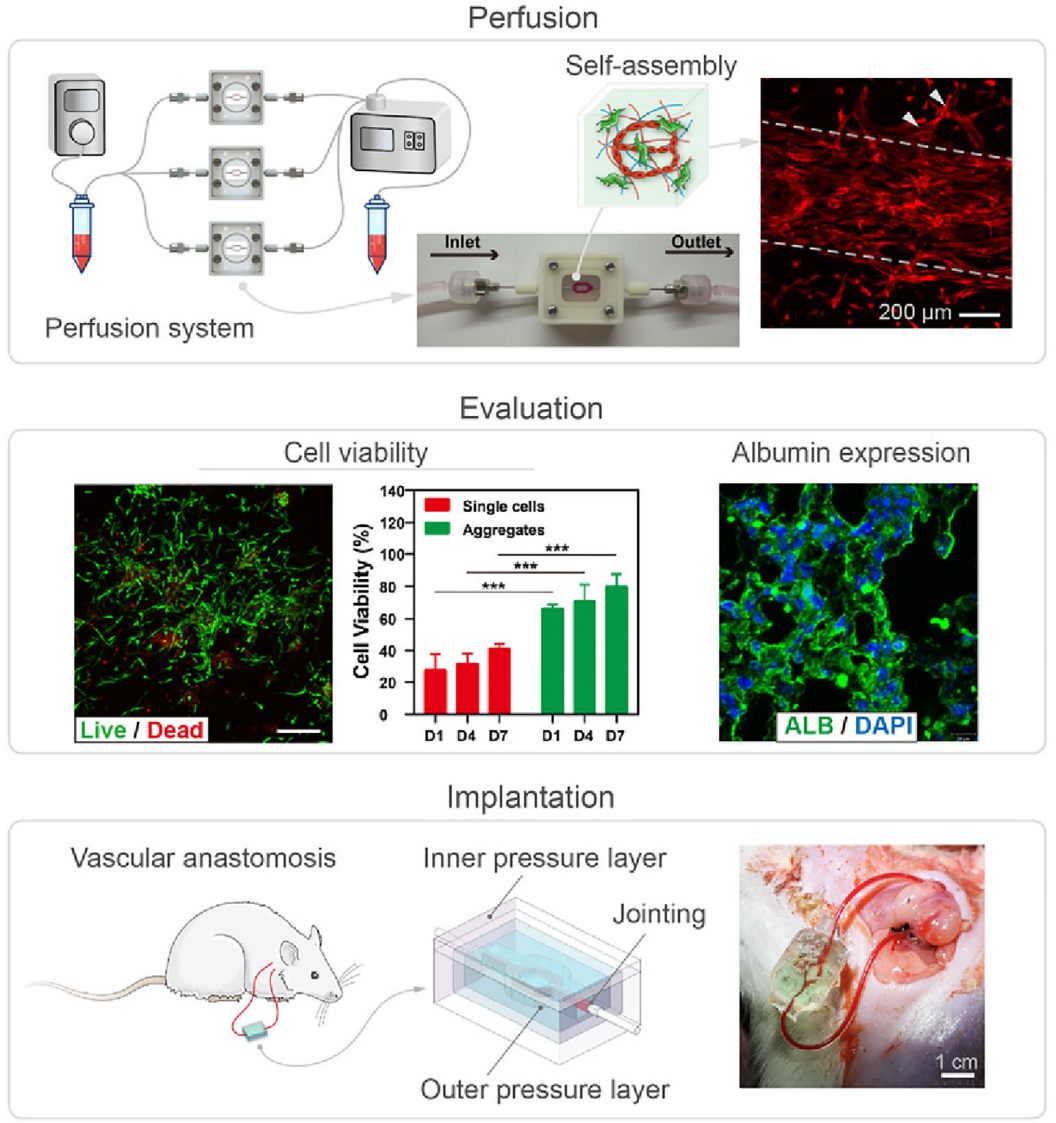
Process to realize the Regeneration aspect of the RR framework. Reproduced with permission.^40^ Copyright 2021, WILEY.

In the RR strategy, bioprinting only constitutes half of the work. Following bioprinting, long‐term nutrient solution perfusion should be employed to guarantee tissue activity and encourage cell self‐assembly, while culture effects should be quantitatively tested before final implantation into animal models to evaluate tissue function in a real‐world environment (Figure 10).

#### 2.5.1 Perfusion

The in vitro perfusion device emulates the umbilical circulation in utero to facilitate tissue growth and development with three components: (1) perfusate components, (2) fluid control and (3) environmental control. To achieve vascularized tissues, the purpose of perfusion can also be to form a monolayer endothelial tubular wall, akin to physiological conditions. When an EC or endothelial progenitor cell (EPC) suspension is perfused into the bioprinted vessels, the cells can adhere to the tubular wall,^94^ due to cell‐adhesive ligands and appropriate flow conditions. While further investigations are necessary to explore artificial organ perfusion culture, we can also take advantage of other systems such as organoids‐on‐a‐chip,^95^, ^96^ in vitro organ maintenance systems,^97^, ^98^ or other in vitro systems under research. Moreover, perfusion culture enables in vitro maturation of tissues, such as targeted differentiation of stem cells, regulation of appropriate WSS during perfusion to stimulate endothelial cell growth and anastomosis of capillary networks. The perfusion medium closest to the in vivo environment is blood, yet currently used serum media or media with known ingredients may also trigger in vitro maturation of printed tissues. It is also possible to directly transplant immature tissue precursors directly into the body for additional maturation under induction of the in vivo environment.

#### 2.5.2 Evaluation

Current quantitative metrics for evaluating tissues during or after culture include (1) cellular activity (e.g., viability,^58^ spatial distribution of cell activity,^56^ MTT intensity field,^18^ cell generated forces^99^), (2) tissue metabolism (e.g., metabolic output^18^), (3) vascular morphology (e.g., maximum invasion depths^30^), (4) vascular mechanics (e.g., burst pressure^100^) and (5) physiological metrics for specific organ types. We can search for suitable physical parameters in well‐established areas of cell biology or physiology. Nevertheless, bioprinting‐based organ manufacturing also has its own peculiarities, such as it differs from simple cell culture and evaluation as it includes interactions between matrix materials and cells in 3D space. Effective transport and assembly processes are vital for the proper functioning of cells. However, even with in vitro constructed tissue components and theoretical models, the physiological functions and morphological structures remain relatively basic and fall short when compared to those found in natural tissues and organs. As a result, the detectable physiological indicators might not match actual physiology, which calls for further research and development.

#### 2.5.3 Implantation

The requirements and methods for implantation have yet to be systematically studied, limiting future applications for pathophysiological models and organ transplantation. Implantation strategies will vary marginally for different tissue types and volumes, but there are typically four aspects to consider. (1) Anastomosis: seamless connection with the blood vessels in the body is essential, especially considering the contradiction between the pressure‐bearing nature of vessels and the need for porosity to enable nutrient penetration.^40^ (2) Circulation adaptation: the main scientific challenge here is to prevent the occurrence of coagulation and thrombosis, and maintain functional stability in circulation over time while ensuring that the biodegradation rate matches the regeneration rate. (3) Functional interactions: considering the intricate two‐way interactions between organ and host, assessing whether the relevant parameters in vitro are still pertinent in vivo is an essential topic in which engineering cybernetics may be beneficial. (4) Immune modulation: a nonspecific immune response can activate angiogenesis, whereas a specific immune rejection, causing a powerful immune reaction, may eventually cause the graft to be rejected.

## 3 RESEARCH PARADIGMS FOR TODAY AND FUTURE

### 3.1 Construction‐comprehension cycle

**FIGURE 11:**
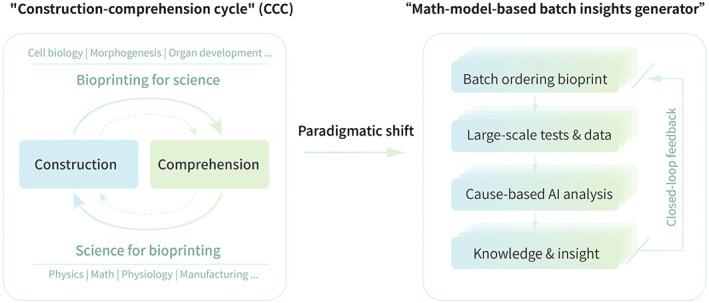
Schematic diagram of two research paradigms in the field of biomanufacturing.

Richard Feynman famously declared, ‘what I cannot create, I do not understand’. During our research, which was supported by the Strategic Priority Research Program (SPRP) of the Chinese Academy of Sciences (CAS), we proposed the ‘Construction‐Comprehension Cycle’ (CCC), thereby forming the ‘Science for Bioprinting, Bioprinting for Science’ research paradigm, which fosters an upward spiralling progression (Figure 11).

1. Science for bioprinting. Organ manufacturing is an archetypal interdisciplinary discipline, and its growth cannot be dissociated from the command and utilization of basic science. For instance, biology furnishes principles or data; physics gives models of physical processes; computer science provides digital models of tissues and organs; material science offers design theories and techniques for inks; engineering assists in establishing bioprinter hardware and software; and so forth.
2. Bioprinting for science. The emergence of bioprinting technology offers an exceptional manufacturing and experimental modelling platform for scientific research. For example, bioprinting can rapidly manufacture intricate 3D cellular microenvironment models or large‐scale structures that can be used to explore cellular behaviours, interactions and morphogenesis, which could generate groundbreaking biomedicine insight and revolutionize fundamental understanding in biology.^101^

### 3.2 New paradigm, new hope

The diminishing returns on investments into biomanufacturing have become a prevalent theme in recent years. For example, the past decade has yielded countless studies on pioneering bioink material development. Nonetheless, many of these studies offer so few groundbreaking insights that it remains unfeasible to design materials in a target‐oriented manner, which has sparked discussions about necessary shifts in the scientific research paradigm (Figure 11). Thanks to advancements in bioprinting technology, batch ordering and experimental mentality can now be employed at the same time. Several printers (e.g., the ‘SIA bioprinter PRO’ we designed) can accomplish extensive batches that cover multi‐factor variables in a single experiment through concentration gradient printing technology, to enable automatic analysis and mathematical modelling. However, despite the fact that big data and artificial intelligence (AI) have become popular research topics in recent years, paradigmatic shifts must be adopted to transition from traditional statistical analysis to causal analysis of multivariate data.^102^ Drawing on the scientific output of the SPRP and other similar research projects in the future, large‐scale tissue and organ manufacturing that integrates biomanufacturing technologies and digital virtual environments could yield new revolutions in biotechnology. In such a paradigm shift, bioprinting technology will play a crucial role in developing new multi‐organ interoperable drugs, uncovering new biological principles and new ‘smart’ regenerative medicines.^103^

## AUTHOR CONTRIBUTIONS

Heran Wang organized the paper framework, presented the main arguments, wrote the full text, and produced the figures and tables. Xin Liu composed the part of 2.3.2, optimized English expressions and reviewed the manuscript. Qi Gu and Xiongfei Zheng guided the idea of the article, proposed many modifications and reviewed the manuscript.

## CONFLICT OF INTEREST STATEMENT

The authors declare no conflict of interest.

## Data Availability

Data sharing is not applicable to this article as no new data were created or analyzed in this study.
